# Genetic testing in a gynaecological oncology care in developing countries—knowledge, attitudes and perception of Nepalese clinicians

**DOI:** 10.1186/s40661-016-0034-5

**Published:** 2016-12-05

**Authors:** Hanoon P Pokharel, Neville F Hacker, Lesley Andrews

**Affiliations:** 1 Department of Obstetrics & Gynaecology, B P Koirala Institute of Health Sciences, Dharan, Nepal; 2 Royal Hospital for Women, Randwick, Australia; 3 School of Women’s and Children’s Health, University of New South Wales, Sydney, Australia; 4 Prince of Wales Hospital, Randwick, Australia

**Keywords:** Physician survey, Genetic testing, Physician attitudes, Nepal, Hereditary gynaecological cancer

## Abstract

**Background:**

Genetic testing for an inherited susceptibility to cancer is an emerging technology in medical practice. Little information is currently available about physicians’ attitudes towards these tests in developing countries.

**Methods:**

We conducted an email survey of Nepalese physicians practicing in academic and non-academic settings in Nepal, regarding knowledge, attitudes and perception towards genetic testing for gynaecologic cancer.

**Results:**

Responses were received from 251 of 387 practitioners (65%). Only 46% of all respondents felt prepared to answer patients’ questions about genetic testing for gynaecologic cancer, despite 80% reporting that patients had asked questions about genetic testing, and 55% being asked more than 5 times in the past year. 42% reported more than 10 of their patients having had genetic testing for cancer, the majority for *BRCA1/2*. Access (40%), cost (37%) and lack of physicians’ information (24%) were cited as the main barriers to testing. The most commonly identified concerns regarding genetic testing were the potential for increased patient anxiety, misinterpretation of results by patients, and maintaining confidentiality of results (64%, 47% and 38% of respondents respectively).

**Conclusion:**

This study shows the gap among the health care providers in developing countries and the available modern scientific tools and skills in regard to the benefits of genetic testing for gynaecological cancers in a developing nation. These findings indicate the need for the introduction of further genetic counselling education and support into gynaecological care in Nepal.

**Electronic supplementary material:**

The online version of this article (doi:10.1186/s40661-016-0034-5) contains supplementary material, which is available to authorized users.

## Background

It is known that a diagnosis of ovarian, fallopian tube, peritoneal or endometrial cancer may be the first indicator of a *BRCA1* or *BRCA2* mutation or Lynch Syndrome due to germline mutations in one of the mismatch repair genes: *MLH1, MSH2, MSH6 or PMS2* [1, 2]. These syndromes account for the majority of inherited gynaecological cancers. *BRCA1/2* mutations account for 14% of all non-mucinous ovarian cancer and 22% of the high grade serous subtype [3]. Evidence of Lynch Syndrome is found in 2% of ovarian cancer cases unselected for age [4, 5] and 9% of endometrial cancer cases under the age of 50 years [2, 6].

**Table 1:** Risk management for an Unaffected Female BRCA1/2 Mutation Carrier

Cancer type	Recommendation	
Breast	Surgical	▪ offer bilateral risk-reducing mastectomy followed by self-surveillance of breast area. The greatest benefit is predicted when surgery occurs at age ≤40 years
		▪ alternatively in the absence of bilateral risk-reducing mastectomy, recommend RRSO preferably around age 40 years
	Surveillance	▪ in families with breast cancer diagnosed under age 35 years, individualised screening recommendations may apply
		▪ otherwise screening should start at age 30 years
		▪ 30–50 years – annual MRI + MMG (+/− US)
		▪ >50 years – annual MMG +/− US
		▪ pregnant - no MRI or MMG, consider US
	Risk-reducing medication	▪ careful assessment of risks and benefits in the individual case by an experienced medical professional is required when considering the use of medication, such as tamoxifen or raloxifene to reduce risk of developing breast cancer in unaffected women. See Cancer Australia Risk-reducing medication resource
Ovarian/fallopian tube	Surgical	▪ recommend RRBSO after family completion or around age 40 years^3^ with peritoneal lavage and close histological examination to exclude occult malignancy
	Surveillance	▪ do not offer serum CA125 and/or transvaginal ultrasound (TVU)
Pancreatic		▪ no evidence of benefit from surveillance

https://www.eviq.org.au Risk Management for an unaffected Female BRCA1 Mutation Carrier

https://www.eviq.org.au Risk Management for an unaffected Female BRCA2 Mutation Carrier

Abbreviations: *RRSO*: Risk-reducing salpingo-oophorectomy, *RRBSO*: Risk-reducing bilateral salpingo-oophorectomy, *US*: ultrasound, *MMG*: mammogram (digital if available), *MRI*: magnetic resonance imaging

**Table 2:** Lynch Syndrome risk management guidelines. All patients should be entered on a local hereditary cancer registry for information and surveillance reminders

Cancer type	Recommendations	
Colorectal	Surgical	▪ consider subtotal colectomy in selected individuals
	Surveillance MSH6/PMS2	▪ annual colonoscopy from age 30 years or 5 years younger than youngest affected if <35 years
		▪ review frequency of colonoscopy at age 60 years with a view to reduced frequency
	Surveillance MLH1/MSH2	▪ annual colonoscopy from age 25 years or 5 years younger than youngest affected if <30 years
		▪ review frequency of colonoscopy at age 60 years with a view to 2nd yearly frequency
	Risk-reducing medication	▪ there may be a reduction of risk in taking aspirin however the appropriate dose is not yet defined (preliminary data)
Endometrial	Surgical	▪ recommend hysterectomy after childbearing complete or from age 40 years, or 5 years younger than the youngest affected, whichever comes first
	Surveillance	▪ there is no evidence for transvaginal ultrasound (TVU) and/or aspiration biopsy
Ovarian	Surgical	▪ recommend risk reducing salpingo-oophorectomy (RRSO) at time of hysterectomy
		▪ recommend HRT at the time of RRSO and continue until the usual time of menopause
	Surveillance	▪ do not offer serum CA125 and/or transvaginal ultrasound (TVU). See Cancer Australia for further information
Gastric	Surveillance	▪ consider second yearly gastroscopy from age 30 years in families with gastric cancer or those at high ethnic risk - e.g. Chinese, Korean, Chilean and Japanese
Urothelial	Surveillance	▪ no evidence of benefit but patients encouraged to report symptoms e.g. haematuria

https://www.eviq.org.au Risk Management for Lynch Syndrome

Identifying mutation carriers now has important implications for the management of these gynaecological cancers, as well as long term surveillance and risk reduction of other cancers. Additionally, at-risk relatives can be offered testing, and appropriate risk management if found to be mutation carriers, or reassured if not. Gynaecologists and gynaecological oncologists have a major role to play in not only identifying women at risk of inherited cancer syndromes and referring appropriate patients to genetic services, but also in managing them appropriately [7].

The issue is much more complex than just referring women for genetic testing and offering them prophylactic treatment. The genetics of hereditary gynaecological cancer is continually evolving and our understanding of the molecular basis of inherited susceptibility to gynaecological cancer has improved considerably [8]. Thus, it is the responsibility of clinicians to keep up to date with advances in this area, so as to support patients to make informed decisions (Tables 1 and 2). A study from Australia revealed that doctors feel it is their duty to inform individuals at risk for hereditary cancer about the availability of genetic counselling [9]. The doctors’ knowledge on the subject, however, seemed to be suboptimal. Indeed, studies have shown that a high proportion of patients do not receive adequate familial cancer risk assessment [10–12]. There has been a steady increase in the availability and application of genetic tests during the past decade [13]. In the USA genetic testing for hereditary cancer is offered by multiple private laboratories. The cost of testing is generally covered by the patient’s health insurer, while in United Kingdom, hereditary cancer genetic testing is covered by the National Health Service. Cancer predisposition genetic testing is not covered by Australia’s national healthcare provider, Medicare, but through the public genetic service ordering the test. Each nation has national guidelines for eligibility for funded testing, with many offering mutation searching where there is an estimated likelihood of finding a mutation of at least 10%.

Genetic testing for susceptibility for gynaecological cancers is widely available in western countries, whereas in developing countries it is still in a rudimentary level. In Nepal National Academy of Medical Sciences, Bir Hospital is in the process of establishing a genetic laboratory. Currently, Nepalese patients are referred to India for genetic testing, where the current cost of *BRCA1/2* testing is approximately US$1400.

The hereditary cancer burden in Nepal is unknown, however a recent study of 50 women with breast cancer diagnosed in Kathmandu found the prevalence of a single mutation (*BRCA1* 185delAG) to be 8%, which is considerably higher than in unselected breast cancer cases in a western population [14], suggesting that hereditary cancer may be just as common, if not more so, than in other populations. Compared with the western developed countries, genetic testing and risk assessment for familial cancer in Asia has been shown to be less available, thus prohibiting the appropriate surveillance, clinical strategies and cancer management of patients and their relatives [15].

Nepal has a population of 27.8 million, and a land area of 147,181 km^2^. The size of the country, relative inaccessibility of mountainous regions and the demographic factors all contribute to difficulties in providing accurate cancer statistics. No population based cancer registry program exists to assess the incidence, prevalence, morbidity and mortality of cancer. The importance of cancer registry data for development of national cancer control programs has been stressed in the context of South Asia [16]. Pooling the cases presented in the main urban centres has been used as a surrogate for a central cancer registry; however these figures are likely to be an underestimate of incidence, as many of the patients go to India or abroad for their further treatment.

A hospital based cancer registry (HBCR) program was started from 1997 in 3 cancer diagnosing and treating hospitals in Kathmandu. Since 2003, with the support of WHO-Nepal, the HBCR program has expanded to cover seven major cancer diagnosis and treatment hospitals in Nepal, which are cooperating to provide relevant data coordinated by the BP Koirala Memorial Cancer Hospital. An initial assessment of incidence at 4 major hospitals found that of 2340 cancers in females overall in 2004, the most common site was cervix uteri (21%), followed by breast (16%), lung (11%) and ovary (6%). However breast and ovarian were the most common cancers amongst women aged 15–34 [17]. This high incidence at young ages raises the possibility of an inherited basis for some of these cancers. In 2012, almost 4000 female cancer cases were registered amongst 7 hospitals, with the most prevalent age group being 50–54 years (12.8%). There was a decrease in cervical and lung cancers, but an increase in breast cancers [18].

There is currently no specialised hereditary cancer service. The level of knowledge of hereditary cancer amongst practitioners seeing women with gynaecological cancer in Nepal is unknown. It is also unknown if these practitioners are currently referring women for genetic testing, and if so, to where. The present study is the first of its kind to be conducted among the Nepalese practitioners regarding the awareness and knowledge about genetic testing of patients with gynaecological malignancy diagnosis.

## Method

A self-administered 15 min survey consisting of 23 questions was designed after a focus group discussion among doctors working at the B.P. Koirala Institute of Health Sciences (BPKIHS) and a literature search of similar topics (Additional file 1). The questionnaire included demographic characteristics, physician practice parameters of speciality, patients seen per week, years of practice and practice setting.

Questionnaires were emailed to 387 general practitioners and specialists of government and private hospitals in Nepal. Their fields included Gynaecologic Oncology, General Gynaecology, Internal Medicine, Family Medicine, Community Medicine/Public health and Primary Care. Practitioners were identified through the Nepal Society of Obstetricians and Gynaecologists and the faculties of the B.P. Koirala Institute of Health Sciences (BPKIHS).

The questionnaire was sent with a cover letter explaining the aims of the study and reassuring the respondents that their responses would be de-identified. Two reminder emails were sent to non-responders at two-week intervals. The email also gave the option of requesting another questionnaire if the original one had been misplaced/discarded.

Doctors’ consent to participate was inferred by completion and return of the questionnaire.

The study was approved by the UNSW Human Research Ethics Advisory (HREA) Panel, University of New South Wales, Sydney, Project number-HC16248.

## Results

Responses were received from 251 of 387 practitioners (65%) – 118 gynaecologists (47%), 36 gynaecological oncologists (14%) and 97 clinicians from other fields (39%). There was a trend for gynaecologists to be older (36% aged over 50, vs 17% both of gynaecological oncologists and other clinicians) and more likely to be female (72% vs 33% of gynaecological oncologists and 47% of other clinicians), but differences did not reach significance.

**Fig. 1:**
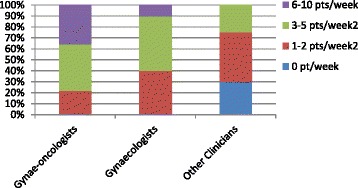
Number of Gynaecological cancer patients seen per week by the clinicians

Overall, half of the respondents saw less than 3 patients per week regarding gynaecological cancer (which included 30% of non-gynaecologists who reported seeing none). No respondent reported seeing more than 10 gynaecological cancer patients per week, with 64% of gynaecological oncologists, 90% of gynaecologists and all clinicians from other fields seeing less than 6 patients per week regarding gynaecological cancer (Fig. 1).

Approximately half of all respondents felt prepared to answer patient’s questions about genetic testing for gynaecologic cancer (46%). However, while 94% of gynaecologic oncologists felt prepared, only 47% of gynaecologists and 26% of other clinicians did so, despite 86% of gynaecologists and 71% of other clinicians reporting that patients had asked questions about genetic testing. Non-gynaecologic clinicians had lower requests for information with a third reporting never being asked, and half being asked on less than 5 occasions in the past year. Gynaecologists and gynaecological-oncologists had similar requests with around 58% of each group reporting less than 5 requests in the past year and 39% and 36% respectively being asked on 6–10 occasions.

All groups demonstrated a bimodal distribution of the number of patients having had genetic testing for cancer predisposition, with overall 40% reporting less than 6 patients having had testing and 42% reporting more than 10 of their patients having had genetic testing for cancer. The majority of tests were for *BRCA1/2* (70%), with 10% for mismatch repair *genes* (Lynch Syndrome) and 22% reporting other genetic tests. 27% of respondents reported that the results never influenced management, 45% said that they did sometimes and 27% said most of the time.

Access (40%), cost (37%) and lack of physicians’ information (24%) were cited as the main barriers to testing. When asked about how genetic test results influenced their patients’ care, preventive surgery was the most cited option (47%) followed by screening (33%), lifestyle changes (31%) and medication (14%). The vast majority of respondents thought that genetic testing was clinically useful. Of the 23 respondents who did not think genetic testing was clinically useful, the majority (*n* = 17) were non gynaecological clinicians. They cited difficulties in interpreting results, failure to affect patient care and patient anxiety as the main reasons. Amongst all respondents, the most commonly identified concerns regarding genetic testing were the potential for increased patient anxiety, misinterpretation of results by patients, and maintaining confidentiality of results (64%, 47% and 38% of respondents respectively). Approximately one quarter of all respondents noted concerns regarding the provision of post-test counselling, the potential for discrimination, the clinical utility of results, and the accuracy of results. However, 91% of all respondents said that if a patient brought genetic test results, it would be likely or very likely to influence their care.

## Discussion

This is the first analysis of Nepalese clinicians regarding genetic testing for gynaecological cancer. Similar to Baars MJ et al. [19] who surveyed Dutch medical practitioners regarding genetic testing in 2005, we had a pleasing response rate of 65%. Our findings should however be viewed in light of several limitations. We have no criteria to estimate if decliners would answer differently to our respondents. The replies are self-reported with no objective measures, and sub group analysis was limited by the sample size.

Our cohort reported low preparedness to answer patients’ questions about genetic testing for gynaecological cancer, particularly from non-gynaecologists. Remaining respondents were primary care physicians (39%), which is consistent with a study conducted by Keating NL et al., [20] where it was emphasized that wider participation by community-based physicians successfully incorporated genetic testing into practice. The trend is seen that with their involvement, chances of early detection care and referral will increase. This will ultimately facilitate and increase the promotion of genetic testing facilities, and expand Gynaecologists’ role in counselling and testing.

Self-reported utilisation of genetic information was high. This cohort has experienced the need for better clinicians’ education and confidence to discuss results. A number of the issues concerning patient anxiety and confidentiality may be improved by more extensive use of genetic counsellors in the testing process. Western models of care include genetic counsellors as a source of assessment and care of patients suitable for genetic assessment, and our findings that less than half of gynaecologists and a quarter of other clinicians feel prepared to answer questions about genetic testing for hereditary cancer, indicate that there is a need for genetic professionals to assist clinicians in Nepal. The limited access to genetic counsellors is unlikely to change with no tertiary institution in Nepal currently offering this course.

Access and cost of genetic testing may not be such significant barriers with the establishment of the planned genetic service in Nepal. Additionally, the ongoing reduction in the cost of testing panels of multiple genes through next generation sequencing, combined with the ease of saliva and cheek swab testing, is anticipated to facilitate Nepalese doctors ordering testing for hereditary cancers in the future.

Our survey of the Royal Hospital for Women in Sydney [21], found that 23% of all gynaecological cancer patients warranted a genetic assessment. At this hospital, a Hereditary Cancer expert attends all Tumour Board meetings and this allows optimal identification of patients requiring genetic assessment.

Almost half of the respondents cited possible misinterpretation of results by patients as a concern, further highlighting a need for genetic counselling expertise.

Until there are data on the prevalence of hereditary gynaecological cancer in Nepal, estimates of the need for improved genetic care need to be based on outside data. A comparison of the BPKMCH 2010–2012 annual report compared with a recent audit of cases from Sydney [21], indicates there are approximately twice as many gynaecological cancer patients seen at BPKMCH compared with the Royal Hospital for Women. If the mutation prevalence is similar to that of Australia, 20 women diagnosed with gynaecological cancer in BPKMCH each year would be found to carry either a *BRCA1/2* or MMR mutation, providing the opportunity for improved care for themselves and their relatives.

We did not enquire about frequency of patients reporting a family history of relevant cancers, as participant recall may not have been robust. A future prospective audit of consultations from a similar cohort would provide further data to assess the need for hereditary cancer services in Nepal.

In Nepal women diagnosed with gynaecological cancer are treated by general gynaecologists and the women who are referred to Nepal Cancer Hospital and Research centre, Bhaktapur Cancer Hospital and B.P. Koirala Memorial Cancer Hospital are treated by Gynaecologists with additional expertise in Gynaeoncology. There are very few specialist gynaecological Oncologists in Nepal.

## Conclusion

This survey highlights clinicians’ concerns about genetic testing for hereditary gynaecological cancer in Nepal, and provides a basis for consideration of measures to improve knowledge and consideration of testing for affected Nepalese women and their families.

## Additional file

Additional file 1: Awareness towards genetic testing for gynaecologic cancer among the medical service providers in Nepal. (DOCX 17 kb)

## References

[1] Lu KH, Schorge JO, Rodabaugh KJ, Daniels MS, Sun CC, Soliman PT (2007). Prospective determination of prevalence of Lynch syndrome in young women with endometrial cancer. J Clin Oncol.

[2] Kulkarni A, Brady AF (2015). Management of Women with a Gentic Predisposition to Gynaecological Cancers. Royal College of Obstetrics & Gynaecologists. Scientific Impact paper..

[3] Alsop K, Fereday S, Meldrum C, DeFazio A, Emmanuel C, George J (2012). BRCA mutation frequency and patterns of treatment response in BRCA mutation-positive Women with ovarian cancer. A report from the Australian ovarian cancer study group. J Clin Oncol.

[4] Rubin SC, Blackwood MA, Bandera C, Behbakht K, Benjamin I, Rebbeck TR (1998). BRCA1, BRCA2, and hereditary nonpolyposis colorectal cancer gene mutations is an unselected ovarian cancer population: Relationship to family history and implications for genetic testing. Am J Obstet Gynecol.

[5] Malander S, Rambech E, Kristoffersson U, Halvarsson B, Ridderheim M, Borg A (2006). The contribution of the hereditary nonpolyposis colorectal cancer syndrome to the development of ovarian cancer. Gynecol Oncol.

[6] Berends MJ, Wu Y, Sijmons RH, Van der Sluis T, EK WB, Ligtenberg MJ (2003). Towards new strategies to select young endometrial cancer patients for mismatch repair gene mutation analysis. J Clin Oncol.

[7] Brown K, Bunting M (2016). Genetics and gynaecological cancer. Genetics.

[8] Beirne J, Irwin G, Mclntosh SA, Harley IJG, Harkin DP (2015). The molecular and genetic basis of inherited cancer risk in gynaecology. Obstet Gynaecol.

[9] Teng I, Spigelman A (2014). Attitudes and knowledge of medical practitioners to hereditary cancer clinics and cancer genetic testing. Familial Cancer.

[10] Murff HJ, Byrne D, Syngal S (2004). Cancer risk assessment: quality and impact of the family history interview. Am J Prev Med.

[11] Meyer LA, Anderson ME, Lacour RA, Suri A, Daniels MS, Urbauer DL (2010). Evaluating women with ovarian cancer for BRCA1 and BRCA2 mutations: missed opportunities. Obstet Gynecol.

[12] Lanceley A, Eagle Z, Ogden G, Gessler S, Razvi K, Ledermann JA (2012). Family history and women with ovarian cancer: is it asked and does it matter? An observational study. Int J Gynecol Cancer.

[13] Bellcross CA, Kolor K, Goddard KA, Coates RJ, Reyes M, Muin J (2011). Awareness and utilization of BRCA1/2 testing among U.S. primary care physicians. Am J Prev Med.

[14] Bhatta B, Thapa R, Shahi S, Bhatta Y, Pandeya DR, Paudel BH (2016). A Pilot Study on Screening of BRCA1 Mutations (185delAG, 1294del40) in Nepalese Breast Cancer Patients. Asian Pac J Cancer Prev.

[15] Nakamura S, Kwong A, Kim SW, Patmasiriwat PLP, Dofitas R, Aryandono T (2016). Current Status of the management of Hereditary Breast and Ovarian Cancer in Asia: First Report by the Asian BRCA Consortium. Public health genomics.

[16] Bhurgri Y (2004). Karachi cancer registry data - implications for the national cancer control program of Pakistan. Asian Pac J Cancer Prev.

[17] Pradhananga KK, Baral M, Shrestha BM (2009). Multi-institution hospital-based cancer incidence data for Nepal: an initial report. Asian Pac J Cancer Prev.

[18] Pun CB, Pradhananga KK, Siwakoti B, Subedi K, Moore AM (2015). Malignant Neoplasm Burden in Nepal - Data from the Seven Major Cancer Service Hospitals for 2012. Asian Pac J Cancer Prev.

[19] Baars MJ, Henneman L, Ten Kate LP (2005). Deficiency of knowledge of genetics and genetic tests among general practitioners, gynecologists, and pediatricians: a global problem. Genet Med.

[20] Keating NL, Stoeckert KA, Regan MM, DiGianni L, Garber JE (2008). Physicians’ Experience with BRCA1/2 Testing in Community Settings. J Clin Oncol.

[21] Pokharel HP, Hacker NF, Andrews L, et al. Changing patterns of referrals and outcomes of genetic participation in gynaecological-oncology multidisciplinary care. Aust N Z J Obstet Gynaecol. 2016. Accepted 17 Aug 2016. doi:10.1111/ajo.12504. 10.1111/ajo.12504 27530527

