# Supplementary material for: Genetic testing in a gynaecological oncology care in developing countries—knowledge, attitudes and perception of Nepalese clinicians
Source: Gynecol Oncol Res Pract. 2016 Dec 5;3:12. doi: 10.1186/s40661-016-0034-5 (PMC5137212; doi:10.1186/s40661-016-0034-5)
Supplement: Additional file 1: — Awareness towards genetic testing for gynaecologic cancer among the medical service providers in Nepal. (DOCX 17 kb) [file 40661_2016_34_MOESM1_ESM.docx]

**Appendix A**

**Awareness towards Genetic Testing for Gynaecologic Cancer among the Medical Service Providers in Nepal**

The purpose of this questionnaire is to assess the awareness, experience, opinions and preparedness to answer patients’ questions regarding genetic testing for gynaecological cancer of primary care physicians’, General Gynaecologists’ and other practising Doctors in Nepal.

Background Information

1. What is your gender?

a. Male

b. Female

2. What is your age?

a. <30

b. 31–40

c. 41–50

d. 51–60

e. >60

3. In which **specialty** are you practising?

a. Gynaecology

b. Gynaecologic Oncology

c. Family medicine

d. Internal medicine

e. Community Medicine/ Public health

f. Primary Physician

g. Other, please specify: _____________

4. How would you describe your work setting (if you have appointments at more than one setting, please answer these questions thinking of your primary institution)? Please circle one.

a. University

b. Academic medical centre or medical school

b. Medical centre not affiliated with a university

c. Community hospital

d. Private practice

e. Primary Health Centre

f. Other, Please specify: _______________________

5. How many years have you been practicing as a physician? (Please specify in whole years, rounding up to the nearest year) ______ Years

6. How many new patients would you see each week regarding gynaecologic cancer?

a. none

b.1-2

c 3-5

d 6-10

e>10

7. Do you feel prepared to answer a patient’squestions about genetic testing for a gynaecological cancer?

a. Yes

b. No

8. Have you heard or read about tests for hereditary gynaecological cancer, such as BRCA 1, BRCA2, Immunohistochemistry for mismatch repair genes to help diagnose Lynch Syndrome?

a. Yes

b. No

9. Have any patients ever asked questions about genetic tests for gynaecologic cancer?

a. Yes

b. No

10. In the past year, how many of your patients asked questions about having a genetic test?

a. None

b. <5

c.6-10

d. 10-20

e. >20

11. How many of your patients have undergone genetic testing related to cancer predisposition?

a. None

b. <5

c. 6-10

d. >10

12. For which genes have your patients been tested?

a. BRCA1/2

b. Mismatch Repair –Lynch Syndrome (HNPCC)

c. Other…Please specify__________

d. Not applicable

13. Has the result influenced your management?

1. Never
2. Sometimes
3. Most of the time
4. Not applicable

14. What do you see as the main barriers to genetic testing related to Gynaecological cancer?

a. Access

b. Cost

c. Lack of patient information

d. Lack of physician information

e. Not relevant to patient care

15. For your patients who brought the results from a genetic test to discuss with you during an office visit during the past year, which aspects of your patient’s care did you change based on the results? Circle all that apply.

a. Screening tests that you offered

b. Medications or doses that you prescribed

c. Lifestyle changes that you recommended

d. Frequency of follow-up appointments scheduled

e. Diagnoses that you made

f. Not sure

g. No aspects of patient care

h. Preventive surgery

i. Other aspects of patient care, please specify______________

**j. Not applicable**

16. Did you refer any patient to a specialist based solelyon the results of a genetic test?

a. Yes

b. No

**c. Not applicable**

17. To **whom** did you make a referral? Circle all that apply.

a. Genetic Counsellor

b. Geneticist

d. Medical Oncologist

e. Gynae-Oncologist

f Gynaecologist

i. Other, please specify: _____________________

**h. Not applicable**

18) In general, do you think genetic testing is currently clinically useful for the patient or her relatives, meaning you would take a patient’s test results into consideration when formulating your medical management plan (e.g. when to refer for screening tests, when to undertake prophylactic salpingo-oophorectomy or mastectomy ,when to refer to a specialist, etc.)?

a. Yes

b. No

**If “Yes” please go to question 20.**

19) If no, why do you feel genetic testing is **not** clinically useful? Please, circle all that apply.

a. It is too difficult to interpret what the results mean regarding patient care

b. I would not change a patient’s management based on genetic testing

c. It will cause more patient anxiety

d. Guidelines for management are too difficult to access or follow in our health system.

e. Other, please specify: ________________________

20) If yes, how clinically useful do you feel genetic testing currently is? Please circle one.

a. Very useful

b. Useful

c. Somewhat useful

d. Not useful

21) Which of the following do you see as a clinical benefit of genetic testing? Circle all that apply.

a. Offering screening tests (e.g. mammograms, colonoscopies, and ultrasonography)

more frequently to individuals who are found to be at increased risk

b. Offering screening tests (e.g. mammograms, colonoscopies, pelvic USG) at an earlier age to individuals who are found to be at increased risk

c. Changing medication doses

d. Prescribing medication

e. Recommending lifestyle changes

f. Changing the frequency of follow-up appointments

g. Making a diagnosis

h. Prophylactic surgery

i. Other, please specify: _______________________

22) Which of the following concerns you about genetic testing? Circle all that apply.

a. I do not have any concerns about genetic testing

b. The analytical validity, or accuracy, of the test

results is questionable

c. The clinical utility, or ability to use the results in

practice, is questionable

d. Counseling provided by the clinicians following

genetic testing is inadequate

e. Patients may interpret the results incorrectly

f. Advertisements may mislead patients

g. Results could lead to discrimination in employment

h. Results could lead to discrimination in health

insurance

i. Genetic information may not be kept confidential.

j. Results could increase patient anxiety

k. Physicians may feel obligated to refer patients to specialists, perhaps unnecessarily

l. Physicians may feel obligated to refer patients for follow-up procedures, perhaps unnecessarily

m. Other, please specify: ______________________

23) If a patient were to bring the results from a genetic test to discuss with you during an office visit today, how likely is it that the test results would influence your care of the patient? Please circle one.

a. Very likely

b. Likely

c. Unlikely

d. Very unlikely
